# Acute and Chronic Insomnia: What Has Time and/or Hyperarousal Got to Do with It?

**DOI:** 10.3390/brainsci10020071

**Published:** 2020-01-29

**Authors:** Ivan Vargas, Anna M. Nguyen, Alexandria Muench, Célyne H. Bastien, Jason G. Ellis, Michael L. Perlis

**Affiliations:** 1 Department of Psychological Science, University of Arkansas, Fayetteville, AR 72701, USA; atn010@uark.edu; 2 Department of Psychiatry, University of Pennsylvania, Philadelphia, PA 19104, USAmperlis@upenn.edu (M.L.P.); 3 School of Psychology, Laval University, Quebec, QC G1V 0A6, Canada; celyne.bastien@psy.ulaval.ca; 4 Northumbria Center for Sleep Research, Northumbria University, Newcastle NE7 7XA, UK; jason.ellis@northumbria.ac.uk

**Keywords:** insomnia, hyperarousal, diagnostic criteria

## Abstract

Nearly one-third of the population reports new onset or acute insomnia in a given year. Similarly, it is estimated that approximately 10% of the population endorses sleep initiation and maintenance problems consistent with diagnostic criteria for chronic insomnia. For decades, acute and chronic insomnia have been considered variations of the same condition or disorder, only really differentiated in terms of chronicity of symptoms (days/weeks versus months). Whether or not acute and chronic insomnia are part of the same phenomena is an important question, one that has yet to be empirically evaluated. The goal of the present theoretical review was to summarize the definitions of acute and chronic insomnia and discuss the role that hyperarousal may have in explaining how the pathophysiology of acute and chronic insomnia is likely different (i.e., what biopsychological factors precipitate and/or perpetuate acute insomnia, chronic insomnia, or both?).

## 1. Introduction

There are two general, but fundamental beliefs about insomnia that are critically evaluated in this article: (1) Acute insomnia is simply a briefer form of chronic insomnia, and (2) insomnia (regardless of its chronicity) is characterized by a state of hyperarousal. In order to address these issues, the definitions of, and concepts related to, acute and chronic insomnia and hyperarousal are briefly reviewed. Ultimately, it is argued that the distinction between acute and chronic insomnia needs to be the subject of empirical investigation, especially when evaluating the role of hyperarousal in the pathophysiology of insomnia.

## 2. Defining Acute and Chronic Insomnia (Just a Matter of Time?) 

**Acute Insomnia.** Historically, acute insomnia (AI) has not been well defined or precisely delineated in the literature, despite its having been included in multiple classification systems since at least the late 1970s [1,2]. Part of the problem is multiple terms have been used to refer to AI over this time frame. AI has been classified as adjustment insomnia, stress-related insomnia, transient psychophysiological insomnia, symptomatic insomnia, sub-acute insomnia, and sub-chronic insomnia [3,4,5,6,7,8,9,10]. Such variability in terminology has discouraged consensus building, both conceptually and operationally. Another problem has been that the durational criteria for AI has been defined by default; by any time period shorter than the criteria for chronic insomnia (CI). Over the years, CI has been variably defined as more than 1 month [11], 3 months [12], or 6 months [3], and therefore, AI has also been variably defined as shorter than each of these duration thresholds. Still yet another problem, one that also extends to CI, is that AI has not been defined based on quantitative criteria for sleep continuity disturbance (illness severity viz. sleep latency, wake after sleep onset, early morning awakenings, etc.). Finally, it should be noted that the various definitions and nosological classifications for both AI and CI have been made on the basis of consensus opinion and not based on any empirical derivation (e.g., how many consecutive nights must occur until it is unlikely that the insomnia will remit?). 

In recent years, the issue of “what is acute insomnia and how should it be defined?” has been the subject of renewed interest, largely owing to the conduct of several natural history studies [10,13,14] and the publication of one theoretical review dedicated to AI [6]. In the review, three definitions of AI (Diagnostic and Statistical Manual of Mental Disorders [DSM], International Classification of Sleep Disorders [ICSD], and International Classification of Diseases [ICD]) were compared and contrasted. The central role of precipitating factors for AI was highlighted. Life stress was identified as a/the primary precipitant for insomnia based on a review of several etiological models, and a formal definition of AI was proffered. The definition had both qualitative and quantitative criteria, included the delineation of sub-states, and required that a precipitating life event or stress condition be identified. The specific definition put forward was as follows: AI is defined as sleep continuity disturbance (i.e., difficulty initiating and/or maintaining sleep) occurring on at least 3 days per week for anywhere between 1 week and 3 months. The more than or equal to one week duration threshold (3-day minimum) allowed for the likely possibility of non-pathologic insomnia (i.e., insomnia as a normal variation in sleep continuity, presumably occurring in association with less than optimal circumstances for sleep [sleep without adequate homeostatic priming or sleep that occurs at times outside the individual’s preferred sleep phase]). The remaining time frame (>1 week to 3 months) was subdivided into: Acute (>3–14 days), transient (2–4 weeks), and sub-chronic insomnia (1–3 months). It is important to note that this categorization was not empirically based on insomnia but was in line with other mood disorders [6,12,15]. The quantitative criteria applied to the definition of AI were those that are typically adopted for clinical research [13,16]. This rule is generally applied to typical or average sleep latency and/or wake after sleep onset values. More specifically, individuals that take ≥ 30 minutes to fall asleep or who are awake for periods of this magnitude during the night are identified as having ‘difficulty initiating and/or maintaining sleep’ (insomnia of a severity that warrants and/or can be successfully treated). While Ellis and colleagues do not, it is also possible to apply the 30-minute rule to early morning awakenings (awakening 30 or more minutes prior to one’s desired time to awake is deemed problematic). In addition to chronicity, frequency, and severity criteria, it was also stipulated that an AI episode must have a clear precipitant, i.e., that the individual must be able to identify a triggering event characterized as, “(1) any life event or train of life events that results in a significant reduction in quality of life from the individual’s ideal and/or (2) distress at one’s current situation” [6]. The concept that AI must be triggered by an event (a bio-behavioral reaction to stress [perceived or real threat]) is further amplified in the closing section of the review. Here Ellis and colleagues suggest that AI may be conceptualized as part of a larger, and non-pathological, biopsychosocial process. That is, acute insomnia may be considered a normal part of the fight/flight response. Evolutionarily speaking, AI likely represents a necessary override to the normal two process regulation of sleep [17]. That is, if it is not safe to sleep, one should not sleep (regardless of the duration of prior wakefulness and/or time of day). This concept is not new but was presaged by Spielman and Glovinsky in the 1990s when they stated, “No matter how important sleep may be, it was adaptively deferred when the mountain lion entered the cave” [7]. 

**Chronic Insomnia.** In contrast to AI, the literature on chronic insomnia is extensive [18,19]. As would be expected, formal definitions have been developed and adopted, though not without revisions over the last 40 years. Historically (and as noted above), the definition of what constitutes “chronic” has varied greatly. Presently, CI is a diagnostic category within all three of the major nosological systems: ICSD-3 (Chronic Insomnia Disorder [20]), ICD-11 (Chronic Insomnia [21]) and DSM-5 (Insomnia Disorder [12]). All three classification systems include as criteria that the sleep problem is: (1) Characterized by difficulty initiating and/or maintaining sleep; (2) associated with significant daytime consequences, impairment, or distress; and (3) present despite adequate opportunity for sleep. The ICSD-3 and the DSM-5 additionally specify that the sleep continuity problem must occur with a frequency ≥ to 3 days per week and be of a duration of at least 3 months. The ICD-11 does not specify quantitative criteria for insomnia frequency or chronicity and none of the diagnostic systems utilize a quantitative threshold for severity (i.e., adopt the 30-minute rule that is commonly used for clinical research [13,16]). 

The changes in diagnostic criteria, especially with respect to chronicity, have made it difficult to differentiate what constitutes AI versus CI (i.e., when does AI end and CI begin?). Assuming that AI and CI represent different stages of a single disease process, it may be that the common dichotomization is too simplistic and does not adequately capture the clinical course of insomnia disorder. For example, when conducting a natural history study, we observed that not all individuals experiencing AI go on to recover normal sleep or develop CI, some individuals develop a form of persistent poor sleep that does not meet criteria for either AI or CI [14]. This observation has prompted some investigators to denote a third type of insomnia as “subsyndromal insomnia”, “intermediate insomnia”, or “persistent poor sleep” [9,13,14,22]. Finally, it should be noted that AI and CI may not represent different stages of a single disease process; it may be that AI and CI are symptomatically similar but are distinct clinical phenomena (e.g., occur with different frequencies and severities) with distinctly different biological bases. For example, acute insomnia may occur in association with hypercortisolemia [23], while chronic insomnia may occur in association with hyperorexinemia [24] or abnormally low levels of γ-aminobutyric acid (GABA) [25]. 

## 3. Prevalence and Incidence of Acute and Chronic Insomnia

A number of epidemiological studies have been conducted over the course of the last 30 years [26,27,28]. Nearly all of these investigations have focused on the prevalence rate of chronic insomnia. In general, chronic insomnia has been found to occur in 6–10% of the population [26,27,28,29]. Prevalence rates as high as 30% have been reported when: Duration of illness is not taken into account, qualitative criteria are used for insomnia frequency, and/or when daytime impairment criteria are not used to define “caseness” [27,28]. Data on the prevalence of AI has only recently been published [13]. Using the same criteria as outlined in the previous section, the prevalence rates of AI were 9.5% (USA sample) and 7.9% (UK sample). Alternatively, several studies have attempted to estimate the incidence of acute insomnia (typically defined as new onset insomnia). The first such study was undertaken by Ford and Kamerow (1989) [26]. They reported that the incidence rate of new-onset insomnia was 6.2% [26]. A decade later, a similar study was undertaken by Foley and colleagues [30]. They reported that the three-year incidence rate of insomnia was 15% (or an annual incidence rate of approximately 5%). More recent studies provide convergent data suggesting that the annual incidence of insomnia is between 7–8% for insomnia “syndrome” and up to 30% for the occurrence of “insomnia symptoms” [10,31]. The only study to explicitly assess AI (using diagnostic criteria) reported that the incidence rate of AI was between 31–37% (depending on whether DSM criteria with or without additional case criteria (sleep latency or wake after sleep onset of 30 min or longer and a self-reported increase in daytime impairment) was used) [13].

While there is a large measure of consistency among the AI studies (despite different methods, measures, sampling rates, and time frames), the reported incident rates, for most of these studies, were not truly the incidence of acute insomnia but rather the identification of new onset insomnia of any duration (includes both AI and CI). Put differently, most of the incidence studies used interval assessment strategies, where sleep continuity was profiled every one to three months [10,30,31]. This sampling rate lacks the temporal resolution needed to identify the onset and offset of short bouts of insomnia. In order to resolve such a phenomenon, one would need to start with a proband of good sleepers and assess them on a daily or weekly basis. Our group recently undertook such a study [14]. Using these data, it was determined that the annual incidence of AI was 27% and that the annual incidence rate of CI was 1.8%. Of those that developed AI, approximately 72% went on to recover good sleep and 19% developed persistently poor sleep (i.e., these subjects did not recover good sleep but also did not meet duration and/or severity thresholds consistent with DSM-5 Insomnia Disorder). 

In sum, the population prevalence of chronic insomnia is around 10% while up to 30% of the population experiences new onset or acute insomnia on an annual basis. Of those that experience AI, the majority recover, a small minority develop CI, and up to 20% develop an intermediate form of sleep continuity disturbance that does not meet criteria for either AI or CI. The existence of this third group speaks to the need to think more dimensionally about insomnia chronicity and/or the need to create a more pluralistic typology. While this may be done on theoretical grounds, empirically defining what constitutes normal sleeplessness, acute insomnia, persistent poor sleep, and chronic insomnia (and how these stages differ with respect to symptom severity and frequency) will no doubt make clearer how these stages differ with respect to their etiology and pathophysiology. 

## 4. Defining “Hyperarousal” 

When acute, hyperarousal occurs as a triggered psychosomatic response to threat-related stimuli. This response, also known as the “fight-or-flight response”, is characterized by the activation of the autonomic nervous system and adrenal cortex with corresponding increases in adrenergic and hypothalamic–pituitary–adrenal (HPA) axis activity [32,33]. The physiologic consequences of such activation include increased heart and respiration rate, peripheral vasoconstriction, increased perspiration, dilation of pupils, increased muscle tension, and increased glucose release via the breakdown of glucogenic stores [34,35,36]. These responses allow the organism to be optimally resourced (for a brief period of time) to fight or flee. While this type of hyperarousal is obviously adaptive, this level of activation is not typically maintained for extended periods of time (e.g., the average HPA axis stress response is between 45–60 minutes from start to finish [35,36]). Put differently, most human stress systems in the body are self-regulating, which prohibits them from staying “on” for extended periods of time [37,38]. This being the case, it has been hypothesized that a secondary process or system exists that allows the individual to maintain an elevated state of “preparedness” in response to longer-lasting environmental demands, if not overt threats [39,40,41]. This form of “hyperarousal” may consist of a lower-level tonic activation of the fight/flight system [42,43] or the activation of an, as of yet to be identified, alternative system. Consistent with the former point of view, it has been found that individuals who work under chronic stressful circumstances, for example, first responders or military service members, have been shown to exhibit higher levels of HPA and adrenergic activation [44,45], though such activation is of significantly lower magnitude than occurs with fight-or-flight responses [46,47,48]. 

## 5. Hyperarousal in the Context of Insomnia

It is practically axiomatic that insomnia is considered a hyperarousal disorder, i.e., that sleeplessness occurs because of cognitive, and/or somatic, and/or neurophysiologic hyperarousal [49,50,51,52]. The evidence base for this belief, however, is not as large or unequivocal as one might expect. In general, the case for hyperarousal is supported by studies of central and/or autonomic nervous systems and endocrine system activation in patients with chronic insomnia as compared to good sleepers. Examples of central nervous system measures include power spectral measures of high frequency electroencephalogram (EEG) activity [51,53,54], EEG-based evoked response potential measures [55,56], imaging measures of brain glucose or oxygen utilization [57], and/or neuroimaging measures of GABA [25,58,59]. Examples of autonomic measures include heart rate [60], heart rate variability [61], electrodermal activity [62], epinephrine or norepinephrine [63], core body temperature measures [64], and/or metabolic rate measures [65]. Endocrine measures typically include blood- and/or saliva-based measures of cortisol [66,67]. It is important to consider, however, that prior research on the hyperarousal theory of insomnia has mostly observed incremental increases in psychobiological indices (e.g., evening levels in cortisol, core body temperature, glucose metabolism, etc.) in comparison to what is “average”. For example, while prior studies have shown significantly greater MSLT scores in patients with insomnia (suggesting a greater daytime wake drive relative to controls (i.e., greater hyperarousal)), the mean group differences ranged from 2–5 minutes [68,69]. That is, these studies compared differences in patients with insomnia to good sleeper controls (i.e., no insomnia history), but not in comparison to fear states (e.g., subjects experiencing acute environmental threats). Put differently, when differences were observed, it was unclear whether the magnitude of the differences observed represented a level of activation that could preclude sleep or whether the observed tonic activation simply represented one factor, of several, responsible for protracted difficulties with sleep initiation and maintenance [70]. This distinction is important as increases above what is normal or average may be statistically significant, but not necessarily clinically meaningful. One approach to clarifying this issue would be to assess the “hyperarousal” of acute and chronic insomnia (across multiple indices) and to compare such data with experimentally elicited startle or fear responses. In this way, the scale of the arousal could be “biocalibrated” and this would allow for inferences about when “hyperarousal” may or may not account for sleeplessness. Alternatively, another approach is to use more naturalistic studies that estimate the level of hyperarousal required to prohibit sleep. For example, findings from two classical studies on ship engineers during on-call periods showed that high-stress/high-anticipation situations can disturb the subjective and objective quality of sleep [71,72]. 

Three additional important considerations when investigating the association between insomnia and hyperarousal are that (1) daytime indices of hyperarousal may be unrelated to nocturnal indices of hyperarousal (and insomnia may be about one or both), (2) the relative impact of wakefulness at night should be taken into consideration when conceptualizing the effect of hyperarousal, and (3) the association between insomnia and hyperarousal may vary with regard to insomnia type and/or sub-type. With regard to diurnal/nocturnal differences, the neural or hormonal indices that are overactive during sleep (e.g., increases in high frequency EEG activity [51,53]), suggesting a state of hyperarousal) may be separate and distinct from those that are overactive during wakefulness (e.g., increases in glucocorticoids [73,74,75]). Put differently, evidence that supports hyperarousal during one phase (e.g., daytime) or state (e.g., wake), does not necessitate that hyperarousal also be present during other phases/states. With regard to nocturnal wakefulness, it is unclear whether the group differences in hyperarousal observed during sleep, such as increased EEG activity, metabolic rate, or hormonal secretion [49,50], are related to condition (i.e., insomnia disorder) or whether they are related to other factors. It is possible, for example, that these differences observed at night are better explained by time spent awake, and not any insomnia-related disease process. Future work should, therefore, account for the extent to which hyperarousal varies as a function of nocturnal wakefulness, as compared to insomnia diagnosis. Finally, it may be the case that hyperarousal is more characteristic of certain types (e.g., CI with objective short sleep duration phenotype [76]) or sub-types of insomnia (initial, middle, and late insomnia [66]), and, therefore, these differences should be accounted for in future research.

## 6. Hyperarousal May Apply to Acute but not Chronic Insomnia

If acute insomnia is genuinely a part of the fight/flight response (i.e., an override to the normal two-process regulation of sleep timing, depth, and/or duration), it follows that hyperarousal is the mechanism by which adaptive sleeplessness is ensured. That is, under unsafe conditions, sleep is naturally inhibited. In the case of CI, while it is logical that the precipitant for sleeplessness may be the same, it is unlikely given that fight/flight levels of hyperarousal may only be sustained for minutes to hours. Thus, the emergent question is whether chronic hyperarousal (e.g., tonic activation of the autonomic nervous system and adrenal cortex) is sufficient to produce sleeplessness by itself or in combination with other factors. The concern, however, is that past research has, in most cases, assumed this to be true. Recent research has been so focused on identifying *the* marker of hyperarousal in insomnia, its reliance on theory and making theory-driven hypotheses has wavered. That is, what markers of hyperarousal, and in what context, are investigators evaluating and are these markers consistent with the theoretical models regarding the etiology and pathophysiology of insomnia? Let’s take hypercortisolemia as an example. One possible explanation for the pathophysiology of insomnia is that CI results from excessive CNS activation [53,77,78,79,80]. The identified substrates for excessive CNS activation have been related to catecholaminergic function and/or HPA axis dysregulation, in the form of increased cortisol secretion [81]. Theoretically this seems plausible given what was discussed above regarding the acute sleeplessness that naturally occurs in response to a perceived or real threat. The scientific literature on hypercortisolemia as an index for hyperarousal in chronic insomnia is surprisingly mixed [66,73,82,83,84,85,86,87]. The primary issue here is that the definition of hypercortisolemia has been inconsistent across studies [67]. Like most other biological systems, the HPA axis is dynamic, with multiple regulatory inputs [88,89], and, therefore, no two measures of HPA axis functioning (or the resultant cortisol secretion) are the same. For example, cortisol secretion at night while one is asleep has a very different regulatory function than the subsequent cortisol awakening response (CAR) [90]. Comparisons between these two indices, in the context of insomnia, are, therefore, difficult to interpret. The overarching point is that our measures of hyperarousal must be theoretically derived. It is not enough to implicate a biological system in the pathophysiology of insomnia and then measure its association using established methods, but without consideration to whether those methods are consistent with theory (e.g., there’s minimal conceptual rationale for observing group differences in CAR among patients with CI) [74,91].

All this said, a conservative reading of the behavioral models of insomnia suggest that acute hyperarousal is sufficient to precipitate acute insomnia but that chronic hyperarousal is not sufficient to serve as a perpetuating factor for chronic insomnia. The behavioral models (i.e., the 3P and 4P renditions) suggest that chronic insomnia occurs largely in association with behavioral factors (e.g., sleep extension or being awake for extended periods of time in the bed and bedroom) and that with time insomnia occurs as “conditioned” wakefulness. Thus, it may be the case that AI is precipitated by hyperarousal and that CI is perpetuated, to some extent by tonic activation of the fight/flight system, but in larger measure by conditioned wakefulness and/or the failure to inhibit wakefulness [92]. If true, it would be expected that AI would show dramatic elevations on measures of sympathetic activation, while CI would only show modest elevations as compared to what is typical of good sleep. 

*Conditioned wakefulness is related to poor stimulus control, or the act of engaging in nonsleep-related behaviors in bed (or the bedroom) or being awake while in bed for extended periods of time. These behaviors result in conditioned wakefulness, which refers to the notion that the induction of the physiology of wakefulness, for example, during the anticipation of sleep, is due to an environmental precipitant. An environmentally conditioned stimulus (CS) produces the conditioned response (CR) of insomnia [93,94]. This CR may also occur during sleep, which may explain why individuals with insomnia are quicker to wake up (i.e., the latency to transition out of the physiological state of sleep may be shorter) and experience more prolonged awakenings during the night (i.e., the probability of a brief interruption in sleep to lead to a full-blown awakening is greater). It is possible that there may be a role for hyperarousal here, in that the environmental CS may elicit a conditioned (physiological) stress response (e.g., an acute increase in HPA/adrenergic hormones or pulse) and that this may in turn elicit conditioned wakefulness. It is important, however, to distinguish this from a true fear response, in that this “stress response” is likely not at that level but only incrementally higher than baseline (normal), but enough to elicit wakefulness. Given some perspectives that insomnia is characterized by a hybrid brain state (localized wakefulness during otherwise global sleep) [95,96], it may also be the case that these psychobiological responses are producing localized wakefulness, which may contribute to the high prevalence of sleep-state misperception in patients with insomnia, or the phenomenon that an individual perceives oneself to be awake (retrospectively), despite being classified (polysomnographically) as being asleep. This said, rather than “hyperarousal”, CI may be better characterized by disinhibition of wakefulness or a failure to inhibit wakefulness during times of sleep or a disconnect between systems that are supposed to be online/offline during wake/sleep (i.e., maladaptive conditioning) [92,95].*

## 7. Where to from Here?

The goal of the present theoretical review is three-fold: (1) To provide a clear definition for acute and chronic insomnia, based on the available nosological manuals and the scientific literature, with a special emphasis on the limitations of these definitions; (2) to provide a clear definition for hyperarousal, as it relates to insomnia; and (3) to proffer the perspective that the two beliefs described at the opening of this paper are not necessarily true, or at least, that the relationship between acute and chronic insomnia (and hyperarousal) is more complex than previously believed. This alternative perspective suggests that AI and CI are not necessarily different points in the natural history of insomnia (disorder), but rather that they are distinct states. More, there is no clear evidence to support that the severity and frequency of AI and CI are the same (it may be the case that AI is a brief yet more intense version of CI) and/or that the biopsychosocial concomitants of AI and CI are the same. While the symptom profiles for the two are the same (at least from a theoretical perspective), the etiology and pathophysiology of each are likely very different, and specifically AI may be more about “hyperarousal”, whereas CI may be more related to other processes, such as homeostatic dysregulation and/or conditioned wakefulness. It is possible that these processes are related to traditional indices of hyperarousal. However, we argue here that the use of the term “hyperarousal” may be a misrepresentation of the magnitude of these responses and that the hyperarousal observed in CI may be different than the hyperarousal observed in AI, and importantly, that these two should not be conflated. 

Based on the current state of the science, the following recommendations are offered, with the intention that they could potentially guide future research efforts:

(1) Natural history of insomnia data should be used to define what constitutes the temporal stages of insomnia. Note: It seems likely that such data will need to capture normal variation in sleep continuity, acute sleeplessness, persistent poor sleep, and chronic insomnia (e.g., Insomnia Disorder).
(2) The temporal stages of insomnia should be compared regarding incident frequency and severity (i.e., does sleep continuity disturbance occur on more days per week and/or with greater average severity when the insomnia is normative vs. acute vs. persistent vs. chronic?).
(3) Multi-method, multi-trait type studies of insomnia should be used to define when “acute hyperarousal” (fight-or-fight responding) is and is not solely responsible for the occurrence of difficulties initiating and maintaining sleep.
(4) Experimental assays or protocols should be developed to distinguish between sleeplessness due to hyperarousal and insomnia due to conditioned wakefulness and/or the failure to inhibit wakefulness.
